# A Review of Changing Episode Definitions and Their Effects on Estimates of Diarrhoeal Morbidity

**Published:** 2007-12

**Authors:** Jim Wright, Stephen W. Gundry, Ronán M. Conroy

**Affiliations:** 1 Centre for Geographical Health Research, Department of Geography, University of Southampton, Highfield, Southampton SO17 1BJ, UK; 2 Water and Environmental Management Research Centre, University of Bristol, 83 Woodland Road, Bristol BS8 1US, UK; 3 Department of Epidemiology and Public Health Medicine, Royal College of Surgeons in Ireland, Dublin, Ireland

**Keywords:** Definition, Developing countries, Diarrhoea, Epidemiology, Morbidity

## Abstract

This paper describes how the methodology used for measuring diarrhoeal morbidity has changed over time and assesses how differences in episode definition have affected estimates of diarrhoeal morbidity among children aged less than five years. The episode definition used in 73 studies included in three previously-published literature reviews was identified. In earlier work, a method was developed that adjusts morbidity estimates to take account of differences in episode definition. This adjustment method was applied to the studies identified in these three literature reviews. Episode definitions were better documented and were more consistent in studies published after 1980. Adjusting morbidity estimates to account for definitional differences did not substantially alter the reviews’ conclusions. Diarrhoeal surveillance has steadily improved since 1980, with methodology becoming more consistent between studies and better documented. Although episode definitions have changed over time, the morbidity estimates derived in the three reviews appear robust to these changes.

## INTRODUCTION

Accurate quantification of the burden of diseases enables research efforts and resources to be targeted towards the most widespread diseases (1). The burden of diarrhoeal morbidity is generally measured in terms of episodes (2). Several studies have found that when undertaking surveillance of diarrhoea, differences in episode definition may affect estimates of the burden of the disease (3–6). To overcome these problems, a standard international definition has been proposed for a diarrhoea episode (4); however, it is unclear at present how far this proposed standard has been adopted in epidemiological studies.

This paper explored these issues through a re-examination of three literature-based studies of the global burden of diarrhoeal disease. We assessed the episode definitions used in the studies that form the basis of these three reviews and considered whether these definitional differences could account for the observed morbidity trend.

## MATERIALS AND METHODS

### Literature review

Three studies sponsored by the World Health Organization (WHO) have drawn on published literature to assess the changing patterns of global diarrhoeal morbidity and mortality among children aged less than five years. The first of these studies drew on work published between 1955 and 1980 (7), the second on work published between 1980 and 1990 (8), and the third on work published between 1991 and 2000 (9). The three reviews identified longitudinal studies of diarrhoeal disease and excluded studies that failed to meet certain criteria. These inclusion criteria included the length of the study (at least one year) and the recall period used, but not the episode definition used. Each of the three reviews collated morbidity for all the included studies and then calculated median estimates of morbidity for different world regions. These studies suggest a slight increase in diarrhoeal morbidity since 1980.

The first of these reviews documented the different episode definitions used in the included studies, but the subsequent reviews did not. To assess the possible impacts of differing episode definitions, we re-examined all the studies included in the two later reviews and documented the episode definition used in each of them.

### Adjusting morbidity estimates for definitional differences

We have previously described a method for adjusting morbidity estimates to take into account differences in the episode definition used between studies (3). To develop this methodology, morbidity data from three African countries were collected for children aged 9-32 months using a simple diary that enabled episodes to be defined according to a range of different criteria. For example, the number of loose or watery stools used in the definition could be varied. Based on the observed differences in morbidity resulting from different episode definitions, we then developed an adjustment method using linear regression. For example, if a study estimated morbidity based on an episode definition of four or more loose stools a day, the morbidity estimate would be adjusted upwards to account for the narrow definition used. If the study used seven diarrhoea-free days to define the end of an episode, the morbidity estimate would be further adjusted upwards to account for the merging together of consecutive episodes.

This methodology can be used only on studies that define diarrhoeal morbidity in terms of numbers of loose or watery stools per day. It cannot be used where studies have adopted a local language word as their definition of diarrhoea (e.g. the Shona word ‘*manyoka*’ in Zimbabwe (10)). We applied our adjustment method to the studies included in the three literature reviews that used a definition amenable to adjustment.

The principal author collated the studies included in the two later reviews and documented the episode definition used in each of them. Such definitions had already been collated by the authors of the earliest review. We then recalculated the medians for each region using these adjusted morbidity figures, following the same methodology adopted in the original review articles. Given that the data used for developing the adjustment factors relating to children in the 12-35-month age cohort, we were only able to adjust the morbidity estimates for children in this age range and not the other cohorts aged under five years covered by the reviews.

The data used for developing the adjustment method were imperfect, since they were based on data of seven months’ surveillance rather than data for the whole year. However, they provide an indication of the effect of episode definition on estimates of the burden of disease, and the same methodology could be repeated using more complete surveillance data.

## RESULTS

### Changes in episode definition over time

The number of loose stools used for defining an episode was not documented for three of the 27 studies published during 1991-2000 (11–13), two of the 22 studies published during 1980-1990 (14,15), and eight of the 18 studies of morbidity published during 1955-1980 (16–23). A further four studies from 1991 to 2000 (10,24–26), five from 1980 to 1990 (27–31), and two from 1955 to 1980 (32,33) used definitions that were not amenable to adjustment, such as local language words or the mother's definition. It is, therefore, apparent that far more studies included in the two later reviews documented this aspect of their methodology than those in the earliest review.

**Fig. 1:**
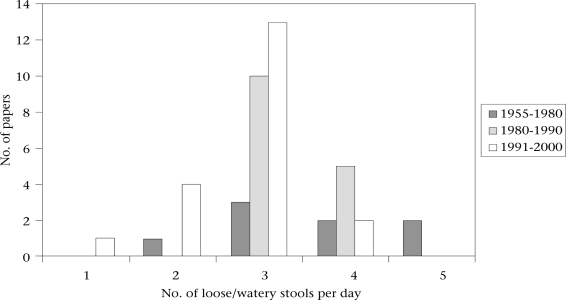
The number of loose or watery stools in 3 literature reviews of studies published during 1955-1980, 1980-1990, and 1991-2000

Figure 1 shows how the number of loose or watery stools used for defining a diarrhoea-day varied for the remaining 20 studies from 1991 to 2000 (34–53), 15 studies from 1980 to 1990 (54–68), and eight studies from 1955 to 1980 (69–76). The proposed standard definition of three or more loose or watery stools per day was more frequently adopted in the two reviews covering studies published during 1980-1990 and 1991-2000 than in the review covering 1955-1980. The number of loose or watery stools used for defining a diarrhoea-day has also decreased slightly over time, averaging 3.8 during 1955-1980, 3.3 during 1980-1990, and 2.8 during 1991-2000.

**Fig. 2:**
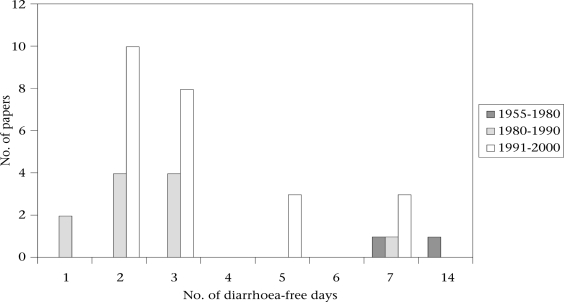
The number of diarrhoea-free days used for defining the end of an episode in 3 literature reviews of studies published during 1955-1980, 1980-1990, and 1991-2000

The number of diarrhoea-free days used for defining the end of an episode was only documented for two of the 18 studies published during 1955-1980 (70,72). This criterion was not documented (14,15,56,59,63,65,67,68) or not a definitional component (27–29) in 11 of the 22 studies published during 1980-1990 and in three of the 27 studies published during 1991-2000 (42,49,50). The proportion of studies documenting this aspect of their methodology thus progressively increased in the later reviews covering the 1980s and 1990s. Figure 2 shows how the number of diarrhoea-free days used for defining the end of an episode varied among the remaining 37 studies. It is somewhat harder to discern trends in this component of the definition, since so many studies published before 1990 did not document this aspect of their methodology. In all the three periods reviewed, only a minority of studies included bloody stools within the definition of a diarrhoea-day.

### Effects of episode definition on global estimates of morbidity

Many studies either did not document the episode definition used, or else used a local definition that was not amenable to adjustment. Most studies included in the earliest review did not fully document the episode definition used. We were, therefore, only able to apply our adjustment method to 15 of the 22 studies (54–68) from 1980 to 1990 and to 20 of the 27 studies (34–53) from 1990 to 2000, where fuller details of the episode definition were available.

**Fig. 3:**
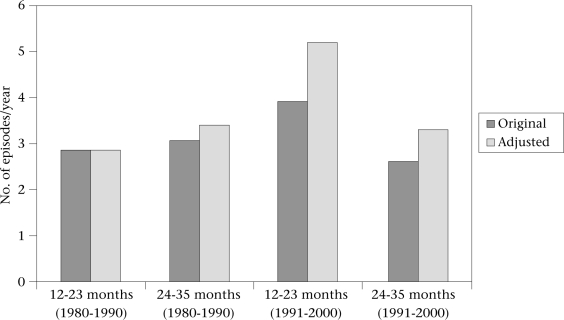
Median age-specific incidence for diarrhoeal episodes per child per year from two reviews covering the period of 1980-1990 and 1991-2000, before and after adjustmnet

Figure 3 shows the estimated global median incidence of diarrhoeal morbidity before and after applying the adjustment factors. To ensure that the original and revised median estimates in Figure 3 were comparable, we recalculated the median incidence from data for all the studies included in the original reviews, whether amenable to adjustment or not. After consulting with one of the review's authors, we established that the median value for the one and two-year age cohorts published in the article by Bern *et al.* had been miscalculated and was incorrect. The unadjusted values in Figure 3, therefore, show the corrected median values calculated from the original data. Although the median number of episodes of diarrhoea is slightly increased by the adjustment method, the trend over time remained the same as that observed in the original paper. In other words, the 12-23-month age cohort showed an increase in the incidence of diarrhoea between the 1980s and the 1990s, with incidence remaining broadly similar in the 24-35-month cohort.

## DISCUSSION

Results of our re-analysis of these earlier literature reviews suggest that the methodological quality of diarrhoeal surveillance has improved over time. Studies are now more likely to adopt an internationally-accepted standard definition for an episode and to document this as part of the publication process. This implies that the potential difficulty of having to combine morbidity estimates based on widely differing definitions is becoming less of a problem.

We were only able to adjust a subset of the reviews’ morbidity estimates to account for differences in episode definition. Several articles used location-specific definitions of an episode, such as the mother's definition or a local language word, which we could not adjust. Such an approach makes survey questionnaires simpler and less confusing to respondents (77) and, therefore, has its advantages. However, while location-specific episode definitions closely match standard medical definitions in some settings (78,79), elsewhere they do not (80). In particular, the differences in prevalence of diarrhoea between Demographic and Health Surveys and Control of Diarrhoeal Diseases Surveys have been attributed to inconsistencies in episode definition and *de facto* use of local language words for diarrhoea (81). This suggests that understanding the relationship between local illness definitions and their standard international equivalents is important where such an approach is adopted.

The adjustment generally increased the original estimates of morbidity. This is because the definition used as a standard (4) included bloody stools, whereas the majority of studies covered by the three reviews did not. Although the adjusted morbidity estimates calculated here were slightly greater than those presented in the original reviews, they do not substantially alter the observed trends in morbidity. This suggests that the methodology adopted in the three reviews is robust to differences in episode definition between the studies included. The original reviews did not attempt to convert morbidity and mortality estimates into disease-burden measures, such as Disability Adjusted Life Years (DALYs), nor have we attempted to do so here. Although only a small adjustment to the reviews’ overall findings was made, it is, therefore, possible that such adjustment may have an impact on the contribution that diarrhoea makes to the global burden of disease.
